# Supplementary material for: CoNiO2/Co3O4 Nanosheets on Boron Doped Diamond for Supercapacitor Electrodes
Source: Nanomaterials (Basel). 2024 Mar 5;14(5):474. doi: 10.3390/nano14050474 (PMC10934051; doi:10.3390/nano14050474)
Supplement: Supplementary file 1 [file nanomaterials-14-00474-s001.zip › nanomaterials-2898450-supplementary.pdf]

Supporting Information

for

$CoNiO_2/Co_3O_4$ Nanosheets on Boron Doped Diamond for Supercapacitor Electrodes

Zheng Cui [1], Tianyi Wang [1], Ziyi Geng [1], Linfeng Wan [1], Yaofeng Liu [1], Siyu Xu [1], Nan Gao [1]*, Hongdong Li [1]*and Min Yang [2]*

[1]*State Key Lab of Superhard Materials, College of Physics, Jilin University, Changchun 130012, PR China*

[2]*College of Chemistry and Life Science, Sichuan Provincial Key Laboratory for Structural Optimization and Application of Functional Molecules, Chengdu Normal University, Chengdu 611130, China*

***Corresponding Authors**

Email address: gaon@jlu.edu.cn (N.G.); hdli@jlu.edu.cn (H.L.); yangmin820525@126.com (M.Y.)

Table S1 Electrochemical property parameters of Co_3O_4 and $NiCo_2O_4$ based electrodes for supercapacitors

electrode	electrolyte	Potential (V)	capacitance	Ref.
Co_3O_4/N-CNO	2 M KOH	0.6	3066 F g^{-1}	[1]
Co_3O_4 nanosheets	3 M KOH	0.4	4127 F g^{-1}	[2]
Copper-doped Co_3O_4	PVA/KOH	0.6	1250 F g^{-1}	[3]
Co_3O_4 sandwich-like	6 M KOH	0.45	1420 F g^{-1}	[4]
$NiCo_2O_4$/graphene	2 M KOH	0.45		[5]
$NiCo_2O_4$ nanosheets	3 M KOH	0.6		[6]
Porous $NiCoO_2$	3 M KOH	0.55		[7]
$NiCoO_2$/Ni	6 M KOH	0.6		[8]
$NiCo_2O_4$@$NiCo_2O_4$	2 M KOH	0.6		[9]
NiO–$NiCoO_2$–Co_3O_4	3 M KOH	0.5		[10]

Table S2 Co/Ni ratio from EDS data

element	Normalized mass (%)	atom (%)
O	23.97	53.71
Co	60.74	36.95
Ni	15.29	9.34

Table S3 Capacitance of diamond-based electrodes for supercapacitors

electrode	electrolyte	capacitance (mF cm^{-2})	Ref.
MnO_2/BDD	1 M Na_2SO_4	7.82	[11]
$Ni(OH)_2$/BDD nanowires	3 M NaOH	91	[12]
BDD/Porous Ti	0.1 M Na_2SO_4	9.55	[13]
3D porous BDD	1 M Na_2SO_4	17.54	[14]
Ni/porous BDD	0.1 MNa_2SO_4	9.55	[15]
porous BDD	1 MNa_2SO_4	17.18	[16]
NBD	1 M KCl	98.9	[17]
TiC/BDD	1 M Na_2SO_4	1.0	[18]
fiber-shaped BDD	1 M Na_2SO_4	8.24	[19]
BDD foam film	0.1 M Na_2SO_4	6.44	[20]

References:

1. Pallavolu, M. R.; Kumar, Y. A.; Mani, G.; Nallapureddy, R. R.; Parvathala, A.; Albaqami, M. D.; Karami, A. M.; Joo, S. W., A novel hybridized needle-like Co_3O_4/N-CNO composite for superior energy storage asymmetric supercapacitors. *J. Alloys Compd.* **2022,** *908*, 164447.

2. Pan, X.; Chen, X.; Li, Y.; Yu, Z., Facile Synthesis of Co_3O_4 Nanosheets Electrode with Ultrahigh Specific Capacitance for Electrochemical Supercapacitors. *Electrochim. Acta* **2015,** *182*, 1101-1106.

3. Liu, S.; Kang, L.; Hu, J.; Jung, E.; Zhang, J.; Jun, S. C.; Yamauchi, Y., Unlocking the Potential of Oxygen-Deficient Copper-Doped Co_3O_4 Nanocrystals Confined in Carbon as an Advanced Electrode for Flexible Solid-State Supercapacitors. *ACS Energy Lett.* **2021,** *6*, 3011-3019.

4. Lai, C.; Guo, Y.; Zhao, H.; Song, H.; Qu, X.; Huang, M.; Hong, S. W.; Lee, K., High-performance double "ion-buffering reservoirs" of asymmetric supercapacitors enabled by battery-type hierarchical porous sandwich-like Co_3O_4 and 3D graphene aerogels. *Adv. Compos. Hybrid Mater.* **2022,** *5*, 2557-2574.

5. Umeshbabu, E.; Rajeshkhanna, G.; Ranga Rao, G., Effect of solvents on the morphology of $NiCo_2O_4$/graphene nanostructures for electrochemical pseudocapacitor application. *J. Solid State Electrochem.* **2015,** *20*, 1837-1844.

6. Yang, J.; Li, H.; He, S.; Du, H.; Liu, K.; Zhang, C.; Jiang, S., Facile Electrodeposition of $NiCo_2O_4$ Nanosheets on Porous Carbonized Wood for Wood-Derived Asymmetric Supercapacitors. *Polymers* **2022,** *14*, 2521.

7. Hu, X.; Wan, C.; Meng, X.; Tang, A.; Ju, X., Porous $NiCoO_2$ nanospheres encapsulated in nitrogen-doped carbon shell achieving high energy storage for aqueous supercapacitors and zinc–ion batteries. *Appl. Surf. Sci.* **2022,** *582*, 152456.

8. Liu, B.; Hou, J.; Zhang, T.; Xu, C.; Liu, H., A three-dimensional multilevel nanoporous $NiCoO_2$/Ni hybrid for highly reversible electrochemical energy storage. *J. Mater. Chem. A* **2019,** *7*, 16222-16230.

9. Wang, X.; Fang, Y.; Shi, B.; Huang, F.; Rong, F.; Que, R., Three-dimensional $NiCo_2O_4$@$NiCo_2O_4$ core–shell nanocones arrays for high-performance supercapacitors. *Chem. Eng. J.* **2018,** *344*, 311-319.

10. Wang, X. W.; Wang, X. E.; Liu, Y. P.; Kong, Y. Y.; Sun, L. Y.; Hu, Y. C.; Zhu, Q. Q., Hydrothermal process fabrication of NiO–$NiCoO_2$–Co_3O_4 composites used as supercapacitor materials. *J. Mater. Sci.: Mater. Electron.* **2017,** *28*, 14928-14934.

11. Yu, S.; Yang, N.; Zhuang, H.; Meyer, J.; Mandal, S.; Williams, O. A.; Lilge, I.; Schönherr, H.; Jiang, X., Electrochemical Supercapacitors from Diamond. *The Journal of Physical Chemistry C* **2015,** *119*, 18918-18926.

12. Gao, F.; Nebel, C. E., Diamond nanowire forest decorated with nickel hydroxide as a pseudocapacitive material for fast charging–discharging. *physica status solidi (a)* **2015,** *212*, 2533-2538.

13. Zhang, J.; Yu, X.; Zhao, Z.-y.; Zhang, Z.; Li, J., Influence of pore size of Ti substrate on structural and capacitive properties of Ti/boron doped diamond electrode. *J. Alloys Compd.* **2019,** *777*, 84-93.

14. Lu, Z.; Huang, N.; Zhai, Z.; Chen, B.; Liu, L.; Song, H.; Yuan, Z.; Zhang, C.; Yang, B.; Jiang, X., Integration of 3D interconnected porous microstructure and high electrochemical property for boron-doped diamond by facile strategy. *Journal of Materials Science & Technology* **2022,** *105*, 26-35.

15. Shi, C.; Li, C.; Li, M.; Li, H.; Dai, W.; Wu, Y.; Yang, B., Fabrication of porous boron-doped diamond electrodes by catalytic etching under hydrogen–argon plasma. *Appl. Surf. Sci.* **2016,** *360*, 315-322.

16. Wang, J.; He, Z.; Tan, X.; Wang, T.; Liu, L.; He, X.; Liu, X. D.; Zhang, L.; Du, K., High-performance 2.6 V aqueous symmetric supercapacitor based on porous boron-doped diamond via regrowth of diamond nanoparticles. *Carbon* **2020,** *160*, 71-79.

17. Guo, T.; Yang, N.; Yang, B.; Schulte, A.; Jin, Q.; Koch, U.; Mandal, S.; Engelhard, C.; Williams, O. A.; Schönherr, H.; Jiang, X., Electrochemistry of nitrogen and boron Bi-element incorporated diamond films.

Carbon **2021,** *178*, 19-25.
18. Xu, J.; Yang, N.; Heuser, S.; Yu, S.; Schulte, A.; Schönherr, H.; Jiang, X., Achieving Ultrahigh Energy Densities of Supercapacitors with Porous Titanium Carbide/Boron-Doped Diamond Composite Electrodes. *Adv. Energy Mater.* **2019,** *9*, 1803623.
19. Zhang, J.; Zhao, Z.; Zhang, Z.; Guo, L.; Xu, L.; Sun, P.; Wang, M.; Gao, M.; Li, Y.; Li, D.; Boukherroub, R., Construction of flexible fiber-shaped boron-doped diamond film and its supercapacitor application. *J. Colloid Interface Sci.* **2023,** *629*, 813-821.
20. Zhang, J.; Yu, X.; Zhang, Z.-Q.; Zhao, Z.-Y., Preparation of boron-doped diamond foam film for supercapacitor applications. *Appl. Surf. Sci.* **2020,** *506*, 144645.

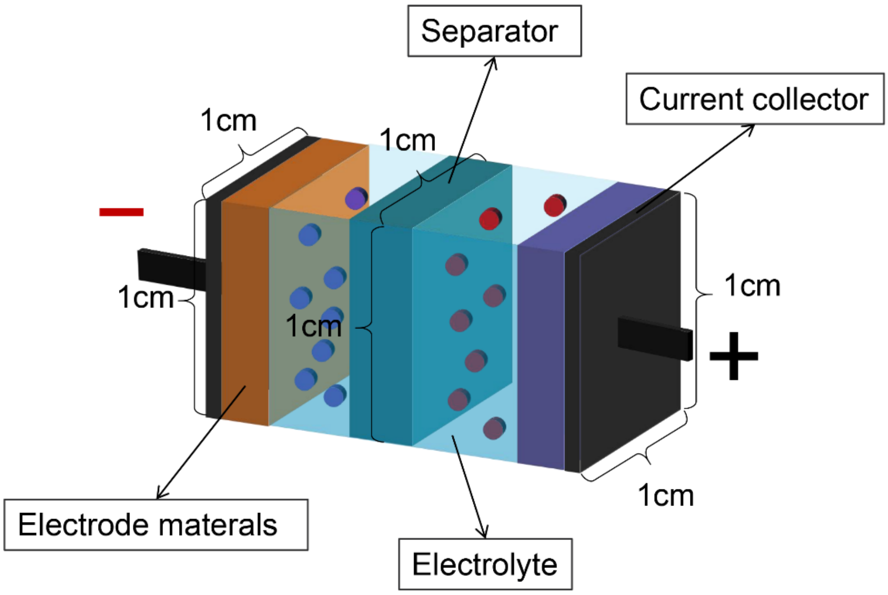

Figure S1 Supercapacitor structure diagram

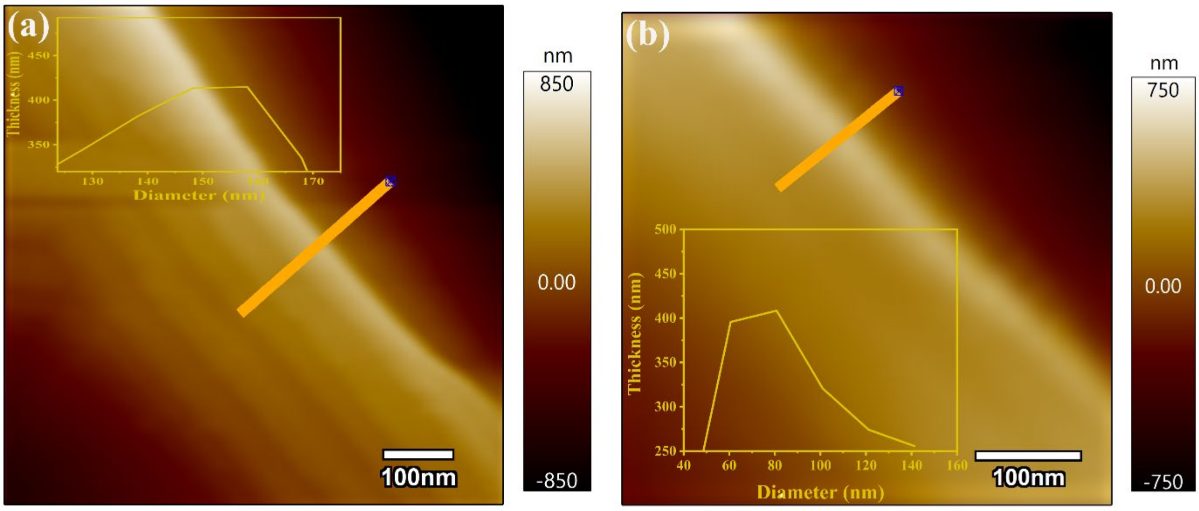

Figure S2 $CoNiO_2/Co_3O_4$/BDD at deposition time of (a) 1000 s, (b) 2500 s the images obtained by AFM image and the corresponding thickness distribution histogram.

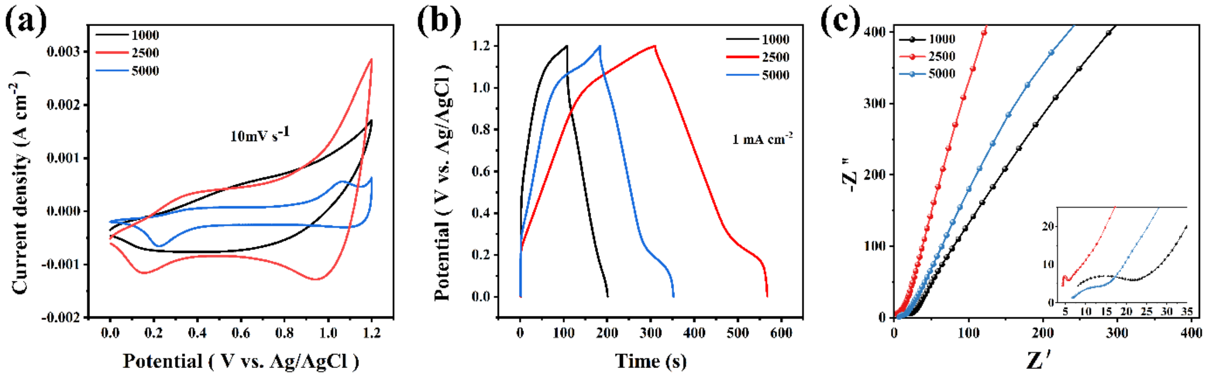

Figure S3 (a) CV, (b) GCD curves and (c) EIS curves of $CoNiO_2/Co_3O_4$/BDD samples with reaction times of 1000 s, 2500 s and 5000 s.

CV curves of $CoNiO_2/Co_3O_4$/BDD electrodes at a constant scan rate of 10 mV·s^{-1} in Figure S1a show that the integrated area of sample with reaction time of 2500 s is apparently larger than others, and it has the longest discharge time (Figure S1b), which is attributed to the fine nanosheet array structure. The Nyquist plots in Figure S1c denote that the slope of the straight line for electrode with reaction time of 2500 s is larger than that of others at the low frequency region, and the lower diffusion resistance indicates fast electric responses during redox reaction.

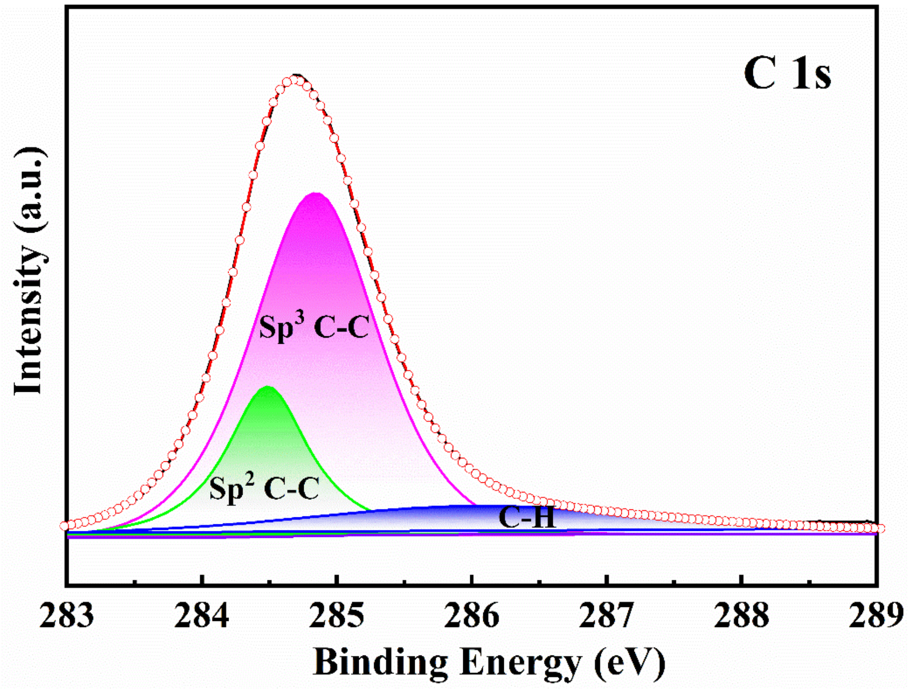

Figure S4. XPS of BDD films.

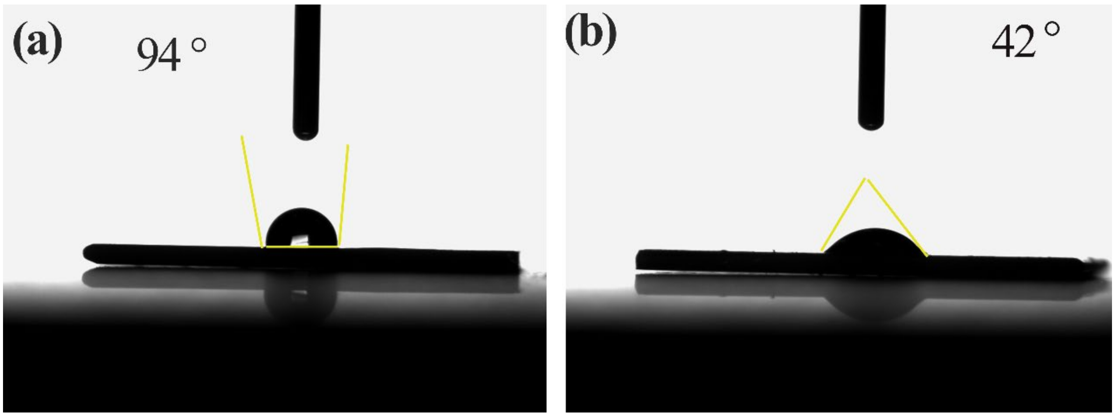

Figure S5 Contact angle measurement of (a) pristine BDD and (b) $CoNiO_2/Co_3O_4$/BDD with 1 mol L^{-1} Na_2SO_4 electrolyte solution.

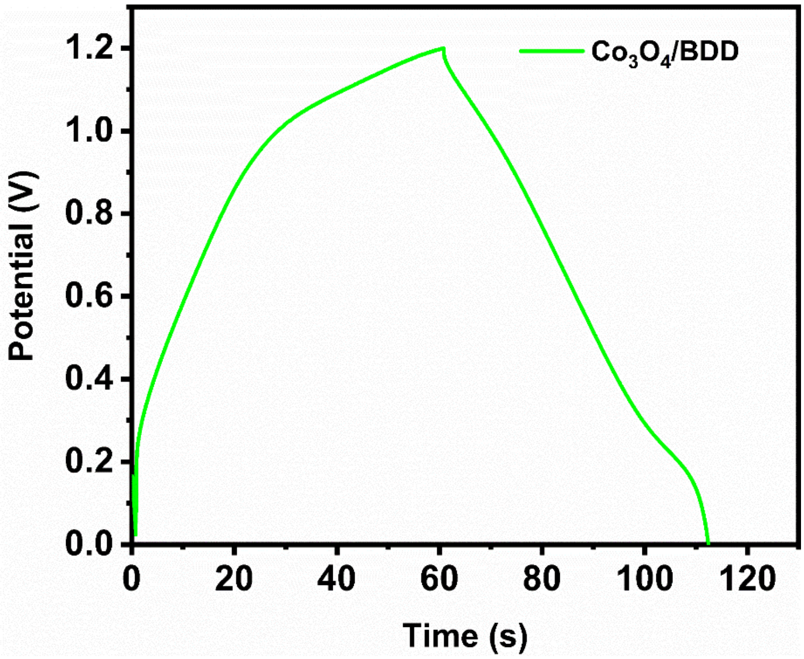

Figure S6 GCD curves at a scanning rate of 2 mA cm^{-2} of Co_3O_4/BDD electrode

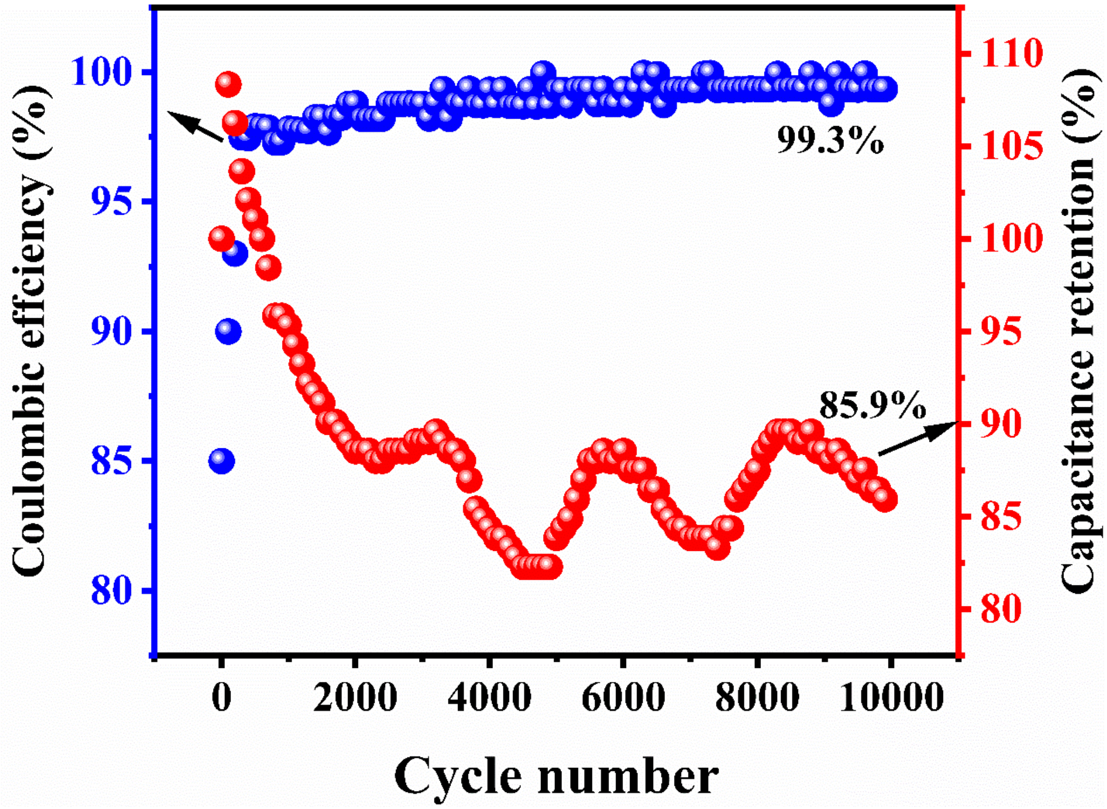

Figure S7 Cycling stability of $CoNiO_2/Co_3O_4$/BDD at 2 mA cm^{-2}.

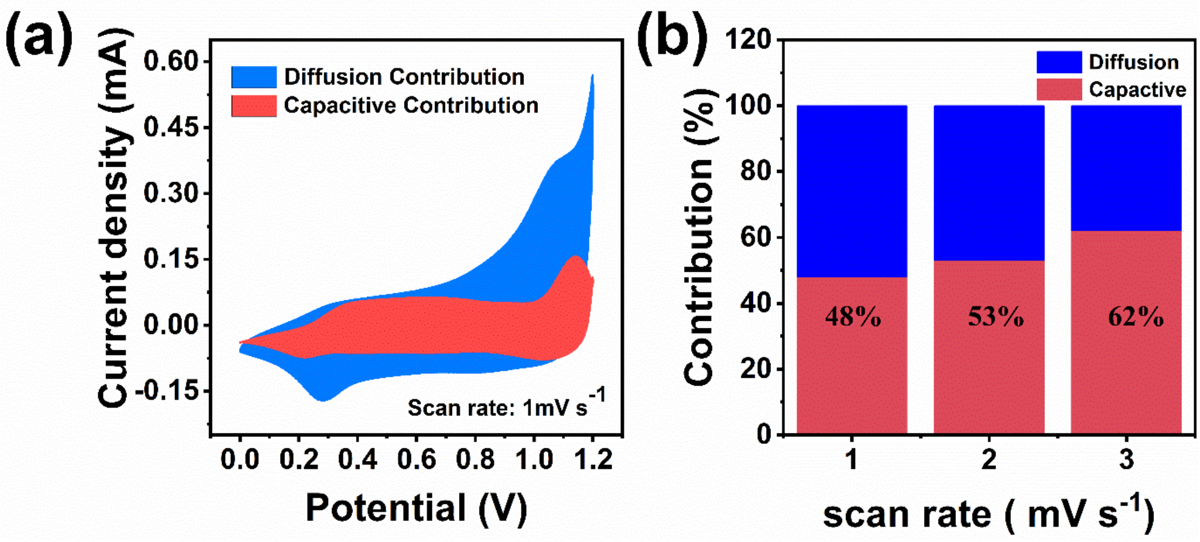

Figure S8 (a) Capacitive and diffusion-controlled charge storage contributions at the scan rate of 1.0 mV s^{-1} (b)The contribution ratio of capacitive and diffusion-controlled charge storage at various scan rates ranging from 1 to 3 mV $s^{-1}$for $CoNiO_2/Co_3O_4$/BDD electrode.

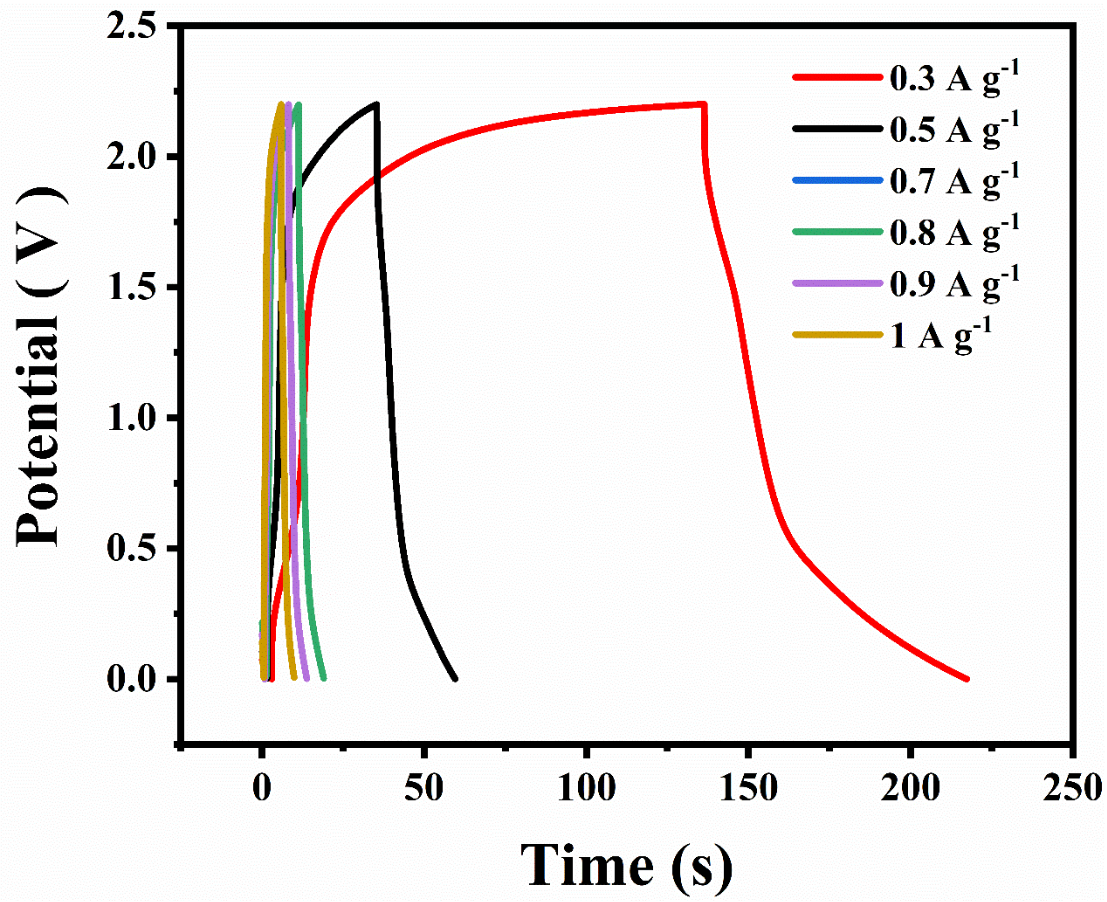

Figure S9 GCD curves at a current density of 0.3 to 1 A $g^{-1}$of the $CoNiO_2/Co_3O_4$/BDD electrode.

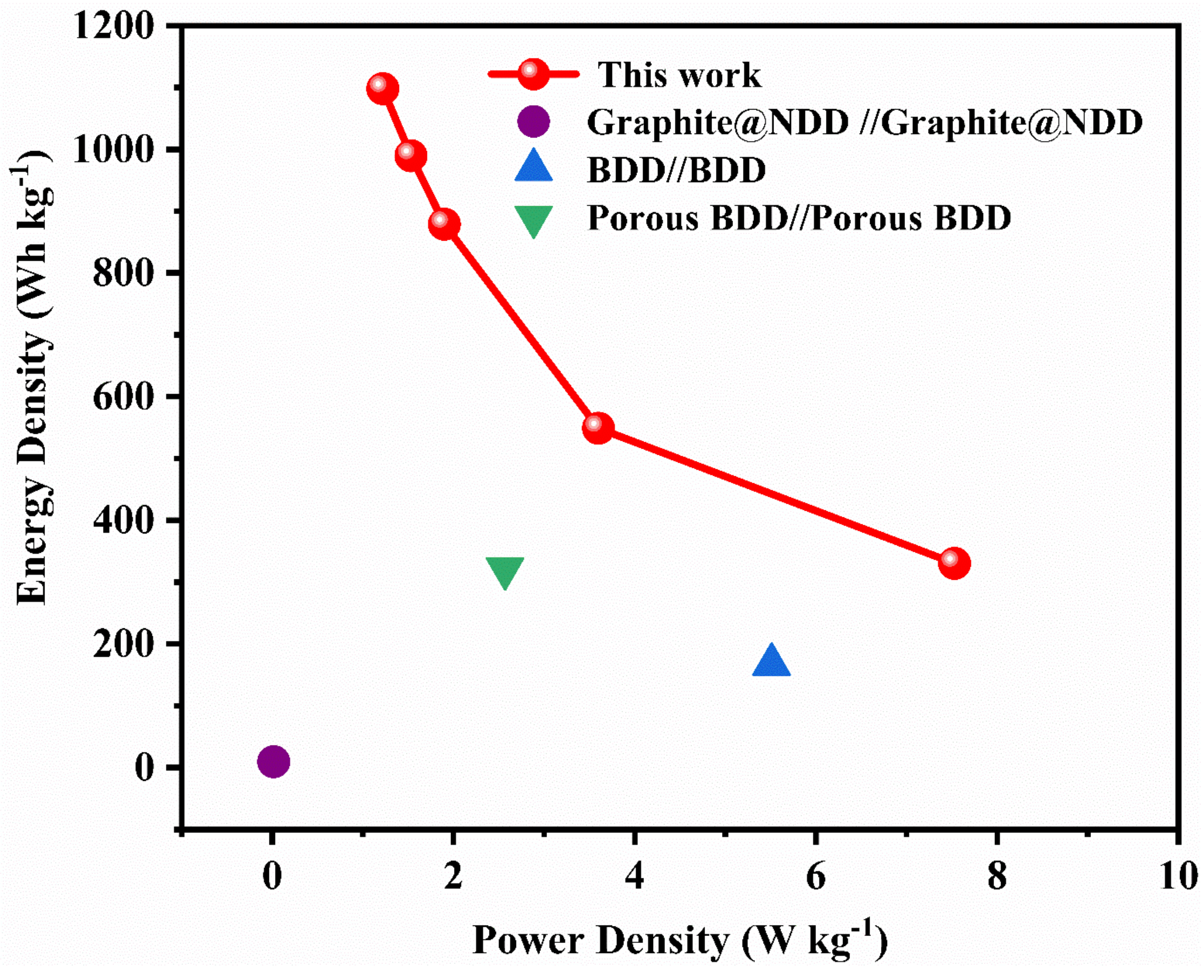

Figure S10 Ragone plot of energy density and power density of $CoNiO_2/Co_3O_4$/BDD and other electrodes obtained from the literature.
